# CALR-TLR4 Complex Inhibits Non-Small Cell Lung Cancer Progression by Regulating the Migration and Maturation of Dendritic Cells

**DOI:** 10.3389/fonc.2021.743050

**Published:** 2021-10-01

**Authors:** Ruo Chen, Min Huang, Xu Yang, Xiao-Hong Chen, Ming-Yan Shi, Zhuo-Fan Li, Zhi-Nan Chen, Ke Wang

**Affiliations:** National Translational Science Center for Molecular Medicine, Department of Cell Biology, Fourth Military Medical University, Xi’an, China

**Keywords:** NSCLC, CALR, DCs, TLR4, immunotherapy

## Abstract

**Background:**

Lung cancer is a common malignant tumor that threatens human life and is associated with high morbidity and mortality rates. Calreticulin (CALR) is a antigen characteristic of immunogenic cell death in non-small cell lung cancer (NSCLC), which is closely related to anti-tumor immunity, but its specific mechanism in anti-tumor immunity remains unclear.

**Methods:**

Immunohistochemical staining was performed to detect the expression of CALR and dendritic cell-lysosome-associated membrane glycoprotein (DC-LAMP) in NSCLC tissues. The cell supernatant was used to induce migration and maturation of dendritic cells (DCs). Western blot and real-time PCR were used to investigate the corresponding molecule expression in the CALR-Toll-like receptor 4 (TLR4)-MyD88 signaling pathway. *In vivo* experiments were conducted to evaluate the role of mCALR in lung cancer progression.

**Results:**

The expression of CALR on NSCLC cell membrane (mCALR) and DC infiltration in NSCLC were positively correlated and were closely related to the prognosis of NSCLC patients. Moreover, mCALR facilitated the migration and maturation of DCs by activating CALR-TLR4-MyD88 signaling and increasing the secretion of TNFα and CCL19, which was inhibited by the loss of TLR4. *In vivo* experiments demonstrated that mCALR inhibited lung cancer progression by facilitating DC infiltration in lung cancer tissues.

**Conclusion:**

Our study explores the function and mechanism of the CALR-TLR4 complex in DC migration and maturation and investigates the inhibitory effect of the CALR-TLR4 complex on lung cancer progression, providing a theoretical basis and ideas for immunotherapy of NSCLC.

## Background

Lung cancer is a common malignant tumor with high morbidity and mortality rates worldwide (1). Although lung cancer mortality has decreased considerably by computed tomographic screening, patients with advanced NSCLC have a low 5-year survival rate and poor quality of life (2, 3). With the development of tumor immunology, NSCLC immunotherapeutic strategies are promising (4). Therefore, it is of great importance to elucidate the immune response mechanism to inhibit NSCLC progression.

Immunogenic cell death (ICD) is a specific variant of regulated cell death, which is driven by pathogens, chemotherapeutics and physical cues, and can induce adaptive immunity against antigens of dead cells (5). In the research of tumor immunotherapy, immunogenic cell death has attracted great attention of researchers, and inducing immunogenic cell death has become a new strategy for anticancer therapy. In response to the inducers of immunogenic cell death, such as doxorubicin or oxaliplatin, tumor cells release the signals of damage-associated molecular patterns (DAMPs) and promote the recruitment and activation of antigen-presenting cells, thereby stimulating anti-tumor immune responses. The immunogenic characteristics of ICD are mainly mediated by DAMPs, which include surface-exposed CALR, secreted ATP and released high mobility group protein B1 (6). Previous studies have shown an interaction between CALR and protein disulfide isomerase family A member 3 (PDIA3) in the endoplasmic reticulum (7, 8). When tumor cells are treated with anthracyclines, the CALR-PDIA3 complex co-translocate from the endoplasmic reticulum to the cell surface, displaying an “eat me” signal and facilitating the recognition and phagocytosis of DCs. Thus, DCs present antigens and fortify the anti-tumor immune response. Blockade or knockdown of CALR suppresses the phagocytosis of anthracycline-treated tumor cells by DCs and abolishes their immunogenicity (9). Therefore, CALR can be identified as a key feature in eliciting an anti-cancer immune response. However, the mechanisms underlying the relationship between CALR and the enhanced ability of DCs remain unclear.

In our study, we showed that mCALR expression was positively correlated with DC infiltration in NSCLC tissues. High levels of mCALR expression and DC infiltration are associated with good prognosis in patients with NSCLC. Moreover, mCALR interacted with TLR4, which increased the expression of TLR4 and the secretion of TNFα and CCL19. These cytokines induce the infiltration and maturation of DCs in tumor tissues, thus provoking anti-cancer immune response and inhibiting NSCLC progression. Our study demonstrates the inhibitory effect of the CALR-TLR4 complex on NSCLC progression and provides a theoretical basis for NSCLC immunotherapy.

## Materials and Methods

### Cell Lines

The cell lines A549 and LLC were obtained from the American Type Culture Collection, and H460 cells were obtained from the Cell Bank of the Chinese Academy of Sciences. All lines were tested and authenticated by short tandem repeat DNA profiling. The cell lines (A549-mCALR, H460-mCALR, and LLC-mCALR) with stable expression of CALR on the cell membrane were constructed by transfection with CALR(del_KDEL)-GPI construct (fused with a Flag tag) (Human: NM_004343; Mouse: NM_007591), which were designed and constructed by GENECHEM (Shanghai, China). The cells were cultured in RPMI-1640 medium at 37°C and 5% CO_2_. The medium was supplemented with 10% fetal bovine serum and 2% L-glutamine.

### DC Induction

PBMCs were collected from healthy volunteers and cultured in AIM-V medium (A3021002, Gibco) at 37°C and 5% CO_2._ Two hours later, cell flasks were gently shaken to suspend and remove the unattached and semi-attached cells, which were cultured to induce immature DCs (iDCs) in AIM-V medium supplemented with 800U/ml GM-CSF (215-GMP-050, R&D Systems) and 500U/ml IL-4 (204-GMP-050, R&D Systems) for 6 days. The supernatant of iDCs was mixed with fresh medium in a ratio of 1:1 to induce mature DCs (mDCs) for 16–18 h with 800U/ml GM-CSF, 500U/ml IL-4, 160ng/ml IL-6 (206-GMP-050, R&D Systems), 5ng/ml IL-1β (201-GMP-100, R&D Systems), 5ng/ml TNFα (210-GMP-100, R&D Systems), and 1μg/ml PGE2 (2296/10, Tocris). The iDCs and mDCs were identified by flow cytometry using PE-anti-human CD80 (305208, BioLegend), PE-anti-human CD83 (305308, BioLegend), PE-anti-human CD86 (305406, BioLegend), PE-anti-human CD14 (367104, BioLegend), PE-anti-human CD11c (301606, BioLegend), PE-anti-human HLA-DR (307606, BioLegend), PE-anti-human HLA-ABC (311406, BioLegend), and PE-anti-human CCR7 (353204, BioLegend) antibodies.

### DC Migration and Maturation Assay

The tumor cells were cultured at 37°C and 5% CO_2_ for 24 hours. For the DC migration assay, the supernatant was collected and added to the lower chambers of the transwell system (24 wells, 8-μm pore size, Corning). A total of 1×10^5^ mDCs were suspended in 300 μl serum-free medium. After 4 hours, the cells in the lower chambers were counted to determine their migration ability. Meanwhile, these invaded cells in NC and mCALR groups were also collected and treated with equal RIPA lysis buffer, respectively. The lysate was collected and mixed with 5× loading buffer. After heating at 100°C for 10 min, the samples were detected by Western blot using anti-β-actin antibody (M1210-2, HUABIO). For the DC maturation assay, the iDCs were suspended in the supernatant collected from tumor cells. After 24 hours, the DCs were collected to detect the expression level of CD83 (PE-anti-human CD83, 305308, BioLegend) using flow cytometry. Meanwhile, anti-CCL19 and anti-TNFα antibodies were used to block CCL19 or TNFα in supernatants from mCALR groups, then DC migration and maturation assay was conducted according to the above methods.

### RNA Extraction and Real-Time PCR

Total RNA was extracted from cell samples and tumor tissues using Total RNA kit II (Omega Bio-tek) according to the manufacturer’s instructions and reverse transcribed into cDNA using the PrimeScript RT Reagent Kit (TaKaRa). Real-time PCR was performed to detect the expression of targeted genes using the TB Green^®^ Premix Ex Taq™ II Kit (TaKaRa). The primers for the target genes are listed in **Table S1**.

### Cell Counting Kit-8 Assay

The cell lines A549-mCALR, H460-mCALR, LLC-mCALR, and their controls (2000 cells) were seeded into 96-well plate with 100 μl RPMI-1640 medium (10% FBS). Each group set up blank control without cells in parallel with experimental group. The 96-well plates were incubated respectively at 37°C under 5% CO_2_ for 0, 24, 48 or 72 hours. According to the manufacturer’s protocol, each well was added 10 μl CCK8 solution (C0005, Topscience) for 2 hours. The absorbance at 450 nm was measured using a microplate reader.

### Cell Wounding Healing Assay

The cell lines A549-mCALR, H460-mCALR, and their controls were seeded into 6-well plate with 2 ml RPMI-1640 medium (10% FBS). When the cell density reaches 80**-**90%, the medium was replaced with RPMI-1640 medium (2% FBS) and the scratches were created by the tip of pipettes. The width of scratches were recorded at 0 and 48 hours.

### Enzyme-Linked Immunosorbent Assay

The tumor cells were cultured at 37°C and 5% CO_2_ for 24 hours, the supernatant was collected to detect the level of TNFα and CCL19 by Human TNFα Precoated ELISA Kit (1117202, DAKEWE) and Human CCL19/MIP-3β ELISA kit (EHC036, NeoBioscience) according to the manufacturers’ protocols.

### Co-Immunoprecipitation Assay

Co-IP assay was performed using the Pierce Co-Immunoprecipitation kit (26149, Thermo Fisher Scientific) following the manufacturer’s instructions. Briefly, anti-TLR4 antibody (sc-293072, Santa Cruz) was used for antibody immobilization, and the lysates and resin were incubated with gentle rocking at 4°C overnight. Finally, the resin was eluted, and flow-through was performed to detect the interacting proteins.

### siRNA Transfection

Gene silencing was performed by transfecting siRNA oligonucleotides (GenePharma) using Lipofectamine 2000 (Thermo Fisher Scientific) according to the manufacturer’s instructions. The siRNA sequences are listed in **Table S2**.

### Isolation of Membrane and Cytoplasmic Proteins

The assay was performed using the Mem-PER™ Plus Membrane Protein Extraction kit (89842, Thermo Fisher Scientific) according to the manufacturer’s protocol. The levels of membrane and cytoplasmic proteins were normalized to the expression levels of NA+/K+-ATPase and GAPDH.

### Western Blot

The cells were treated with RIPA lysis buffer (P0013B, Beyotime) on ice. The lysate was collected and mixed with 5× loading buffer to generate a 1× final solution. After heating at 100°C for 10 min, the samples were loaded onto an SDS-PAGE gel and separated by electrophoresis. Then, the proteins were transferred to PVDF membranes (Millipore), which were incubated with the primary antibodies at 4°C overnight after blocking with 5% non-fat milk. Next, the PVDF membranes were incubated with the corresponding secondary antibodies at room temperature for 1 h. Finally, the images were obtained and analyzed using the Image Lab software (BIO-RAD). The antibodies used in the assays were the following: anti-GAPDH antibody (R1210-1, HUABIO), anti-Flag antibody (M1403-2, HUABIO), anti-CALR antibody (sc-166837, Santa Cruz), anti-TLR4 antibody (sc-293072, Santa Cruz), anti-TNFα antibody (17590-1-AP, Proteintech), anti-CCL19 antibody (MA5-23833, Invitrogen), anti-p65 antibody (10745-1-AP, Proteintech), anti-p-p65 antibody (3033s, Cell Signaling Technology), anti-JNK antibody (9252s, Cell Signaling Technology), anti-p-JNK antibody (9251s, Cell Signaling Technology), anti-p38 antibody (ET1602-26, HUABIO), anti-p-p38 antibody (ER1903-01, HUABIO), anti-ERK1/2 antibody (ET1601-29, HUABIO), anti-p-ERK1/2 antibody (4370s, Cell Signaling Technology), anti-MyD88 antibody (sc-74532, Santa Cruz), anti-NA^+^/K^+^-ATPase antibody (sc-21712, Santa Cruz), goat anti-mouse IgG (H+L) secondary antibody (31430, Thermo Fisher Scientific), and goat anti-rabbit IgG (H+L) secondary antibody (31460, Thermo Fisher Scientific).

### Gene Expression Profiling Interactive Analysis (GEPIA)

GEPIA (10) is a web server for analyzing RNA-Seq expression data of tumors and normal samples (http://gepia.cancer-pku.cn/), which is developed by Peking University. The expression of CALR and TLR4 in NSCLC tissues and normal tissues was analyzed using GEPIA.

### Kaplan Meier Plotter

The Kaplan Meier plotter (http://kmplot.com/analysis/) is an open source software that can be used to assess the effect of different genes on survival in several cancers (11). Overall survival rates of patients with high and low TLR4 expression were analyzed using Kaplan-Meier Plotter and calculated by log-rank test.

### Tumor Immune Estimation Resource Analysis

TIMER (12, 13) (https://cistrome.shinyapps.io/timer/) is a web server for the comprehensive analysis of tumor-infiltrating immune cells in diverse cancer types. The correlations between TLR4 expression and CD8+ T cell, CD4+ T cell, and DC immune infiltration were assessed using TIMER on NSCLC sample data, and the correlations between TLR4 expression and TNFα or CCL19 expression in NSCLC were analyzed using TIMER. In addition, the association between clinical outcome and abundance of DC infiltration was determined using TIMER.

### The Cancer Immunome Atlas Analysis

TCIA (14) (https://tcia.at/home) provides comprehensive immunogenomic analysis of next-generation sequencing data from The Cancer Genome Atlas and other datasets, which was developed and maintained at the Institute of Bioinformatics. Using TCIA, we analyzed the impact of activated DC infiltration on the survival of NSCLC patients.

### Immunohistochemical Staining

Immunohistochemical staining of mCALR and DC-LAMP was performed using two serial NSCLC tissue microarrays purchased from the Shanghai Biochip Company (Shanghai, China). The basic information of tissue samples has been described in detail in Table 6 of our previous study, including gender, age, tumor sizes, survival status, survival time, lymph node metastases, AJCC stages, and pathological grades (7). Briefly, the slices were dewaxed, followed by antigen retrieval with 10 μmol/L citrate buffer at pH6.0. After treatment with 3% hydrogen peroxide-methanol, the sections were incubated with blocking serum for 30 min and the corresponding primary antibodies (anti-CALR antibody, sc-166837, Santa Cruz; DC-LAMP Antibody (104G4), DDX0190P-100, NOVUS) at 4°C overnight. Then, a streptavidin-peroxidase kit and 3,3′-diaminobenzidine (Zhongshan Jinqiao Co., Beijing, China) were used to detect the target proteins. Nuclei were detected using hematoxylin staining. The sections were evaluated by two independent pathologists. The intensity and density of positive cells were two indices to display the expression of CALR protein, according to previous methods (7). The number of DC-LAMP+ DCs was also calculated and classified as high or low according to the median.

### Mouse Experiments

Animal experiments were conducted in strict accordance with the use and care of laboratory animals. The methods used in this study were approved by the Institutional Animal Care and Use Committee of Fourth Military Medical University. Female C57BL/6J mice (6–8 weeks) were obtained from Beijing Vital River Laboratory Animal Technologies. LLC-NC or LLC-mCALR cells (1 × 10^6^ cells in 100 μl medium) were injected subcutaneously (LLC-NC, n=5; LLC-mCALR, n=5). Two weeks later, the tumor masses were removed from the mice. The size and weight of the tumor masses were detected and compared between the mCALR and control groups. The expression of TLR4, TNFα, and CCL19 in tumors was determined using real-time PCR. The relative percentages of CD11c+, CD40+, CD80+, CD86+, MHCII+, CD11c+CD8a+, CD11c+CD8a-, CD11c+CD103+, CD11c+CD207+, and CD11c+CD317+ DCs in tumors were detected using flow cytometry using FITC anti-mouse CD11c (117306, BioLegend), PE anti-mouse CD8a (100708, BioLegend), PE anti-mouse/human CD207(Langerin) (144204, BioLegend), PerCP/Cyanine5.5 anti-mouse CD103 (121416, BioLegend), APC anti-mouse CD317(BST2, PDCA-1) (127016, BioLegend), PerCP/Cyanine5.5 anti-mouse I-A/I-E (107626, BioLegend), PE anti-mouse CD80 (104708, BioLegend), APC anti-mouse CD86 (105012, BioLegend), and PE/Cyanine7 anti-mouse CD40 (124622, BioLegend).

### Statistical Analysis

All data were analyzed using GraphPad 8.0. Significant differences were analyzed using an unpaired Student’s *t*-test or unpaired t test with Welch’s correction with a two-tailed distribution. The correlation between the expression levels of mCALR and DC-LAMP was determined using Spearman’s correlation analysis. Overall survival was calculated using Kaplan-Meier analysis and log-rank test. Statistical significance was set at *P*<0.05.

## Results

### Expression of mCALR and DC Infiltration in NSCLC Are Positively Correlated and Are Closely Related to the Prognosis of NSCLC Patients

**Figure 1:**
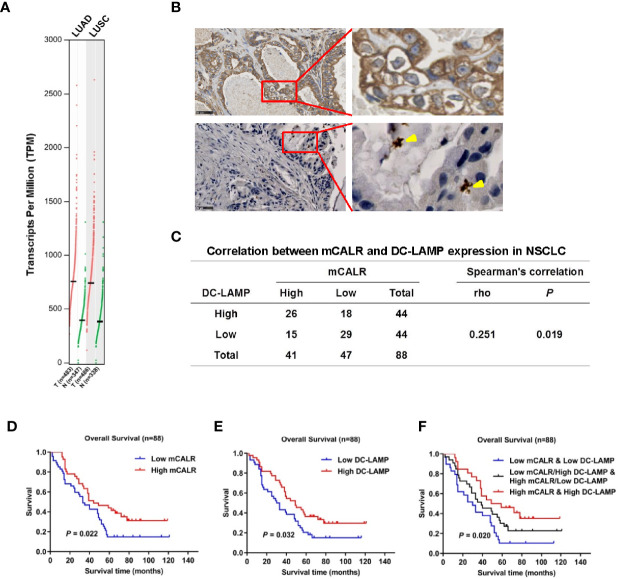
Expression of mCALR and DC infiltration in NSCLC are positively correlated and are closely related with the prognosis of NSCLC patients. **(A)** The expression of CALR in NSCLC (LUAD and LUSC) tissues and normal tissues was analyzed using GEPIA. **(B)** The expressions of CALR and DC-LAMP in NSCLC tissues were determined by immunohistochemical staining; scale bar, 50μm. **(C)** The correlation between mCALR and DC-LAMP expression in NSCLC, 88 samples were evaluated by IHC, high DC-LAMP samples, n=44; low DC-LAMP samples, n=44; high mCALR samples, n=41; low mCALR samples, n=47; Spearman’s correlation=0.251, *P*=0.019. **(D)** Overall survival rates of patients with high and low mCALR; *P*=0.022. **(E)** Overall survival rates of patients with high and low DC-LAMP; *P*=0.032. **(F)** Overall survival rates of patients with combined expression of mCALR and DC-LAMP; *P*=0.020.

CALR has been reported as a potential biomarker in lung cancer (7). GEPIA data showed that CALR was significantly upregulated in lung adenocarcinoma (LUAD) and lung squamous cell carcinoma (LUSC) tissues compared to normal lung tissues (**Figure 1A**). A previous study shows that mCALR on tumor cells displays an “eat me” signal to DCs, which enhances the anti-tumor immune responses (9). To investigate the relationship between the expression of mCALR and DC infiltration in NSCLC tissues, immunohistochemical analyses of mCALR and DC-LAMP, a marker of mDCs, were performed with two serial NSCLC tissue microarrays (**Figure 1B**). The results showed that the expression of mCALR was positively associated with the expression of DC-LAMP (Spearman *r*=0.251, *P*=0.019), which indicated a positive correlation between mCALR expression and DC infiltration in NSCLC (**Figure 1C**). Meanwhile, Kaplan-Meier survival analysis demonstrated that patients with high expression of mCALR (*P*=0.022) and DC-LAMP (*P*=0.032) were significantly associated with good overall survival (**Figures 1D, E**). Because of a positive correlation between mCALR expression and DC infiltration in NSCLC, the combined expressions of mCALR and DC-LAMP were used to predict NSCLC prognosis. The results showed that patients with high mCALR and DC-LAMP expressions had the best prognosis, and the opposite result was obtained in patients with low mCALR and DC-LAMP expressions (*P*=0.020) (**Figure 1F**). All these findings indicate that the expression of mCALR in NSCLC cells is related to mDC infiltration in NSCLC tissues, which displays a good prognosis in patients with NSCLC.

### mCALR Facilitates the Migration and Maturation of DCs by Increasing the Secretion of TNFα and CCL19

**Figure 2:**
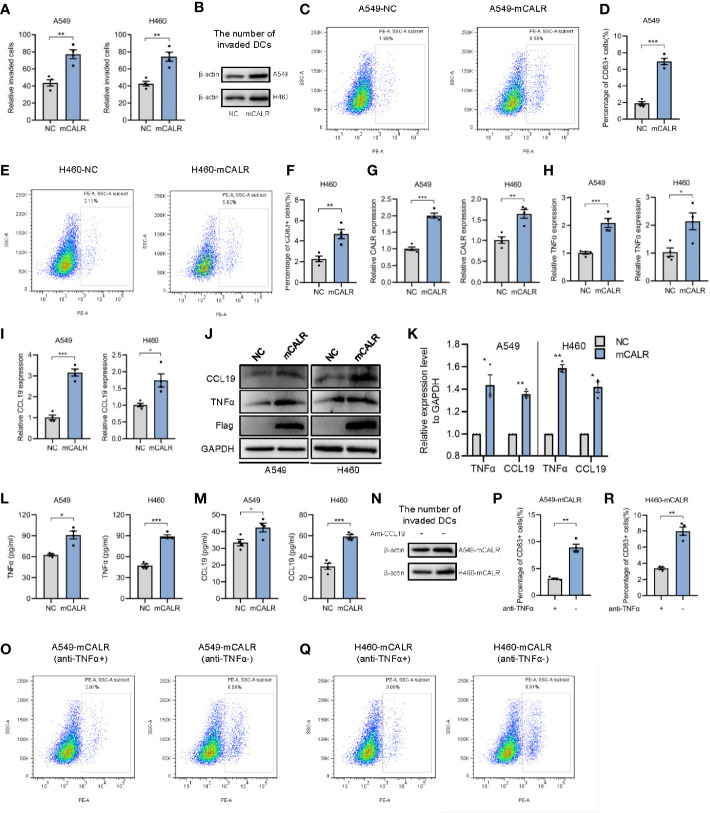
mCALR facilitates the migration and maturation of DCs by increasing the secretion of TNFα and CCL19. **(A)** The effect of cell supernatants (NC and mCALR) on DC migration was evaluated by migration assay; **P < 0.01. **(B)** The number of invaded DCs in both NC and mCALR groups was quantified by western blot using anti-β-actin antibody. **(C–F)** The effect of cell supernatants (NC and mCALR) on DC maturation was determined using flow cytometry with PE-anti-human CD83 antibody; **P < 0.01, ***P < 0.001. **(G–I)** The relative mRNA expression levels of CALR, TNFα, and CCL19 were detected in NC and mCALR groups using real-time PCR; *P < 0.05, **P < 0.01, ***P < 0.001. **(J, K)** The expression levels of TNFα and CCL19 were investigated in NC and mCALR groups using western blot; *P < 0.05, **P < 0.01. **(L, M)** The expression levels of TNFα and CCL19 were investigated in NC and mCALR groups using ELISA; *P < 0.05, ***P < 0.001. **(N)** The number of invaded DCs in both anti-CCL19+ and anti-CCL19- groups was quantified by western blot using anti-β-actin antibody. **(O–R)** The effect of cell supernatants (anti-TNFα+ and anti-TNFα- groups) on DC maturation was determined using flow cytometry with PE-anti-human CD83 antibody; **P < 0.01.

**Figure 3:**
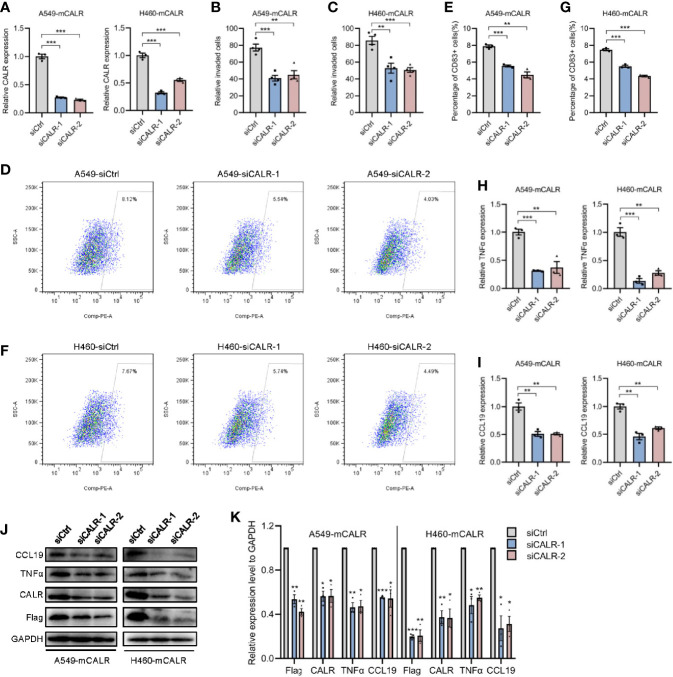
mCALR silence inhibits the migration and maturation of DCs by decreasing the secretion of TNFα and CCL19. **(A)** The relative mRNA expression levels of CALR was detected in siCALR and control groups using real-time PCR; ***P < 0.001. **(B, C)** The effect of cell supernatants (siCtrl and siCALR) on DC migration was evaluated by migration assay; **P < 0.01, ***P < 0.001. **(D–G)** The effect of cell supernatants (siCtrl and siCALR) on DC maturation was determined using flow cytometry with PE-anti-human CD83 antibody; **P < 0.01, ***P < 0.001. **(H, I)** The relative mRNA expression levels of TNFα and CCL19 were detected in siCALR and control groups using real-time PCR; **P < 0.01, ***P < 0.001. **(J, K)** The expression levels of CALR, Flag-mCALR, TNFα, and CCL19 were investigated in siCALR and control groups using western blot; *P < 0.05, **P < 0.01, ***P < 0.001.

On the basis of the foregoing findings, we speculated that mCALR on NSCLC cells might induce the migration and maturation of DCs, causing them to gather around tumor cells and initiate anti-tumor immune response. To verify our hypothesis, cell lines (A549-mCALR and H460-mCALR) with stable expression of CALR on the cell membrane were constructed by transfection with CALR(del_KDEL)-GPI construct and identified by membrane protein extraction (**Figures S1A, B**). CCK8 and Cell wounding healing assay showed that mCALR had no effect on the proliferation and migration of lung cancer cells (**Figures S2A, B**). DCs were also induced from PBMCs according to the protocol shown in **Figure S3A** and identified by flow cytometry (**Figures S3B**). The cell migration assay showed that the supernatant of the mCALR group promoted the migration of mDCs (**Figures 2A, B**). Compared to the control, the percentage of CD83+ DCs significantly increased in the mCALR group after incubation with the supernatant (**Figures 2C–F**). These findings confirm our hypothesis that mCALR in NSCLC cells induces the migration and maturation of DCs. TNFα is commonly used as a DC maturation factor (15), and CCL19 is essential for recruiting CCR7-expressing mDCs (16). Therefore, we investigated the expression levels of TNFα and CCL19 in mCALR cells and controls. The results showed that TNFα and CCL19 levels were significantly elevated at both the protein and RNA levels in the mCALR group (**Figures 2G–K**). The levels of TNFα and CCL19 in supernatant showed a similar result detected by ELISA assay (**Figures 2L, M**). Antibody blockade of CCL19 or TNFα in supernatants from mCALR groups inhibit DC migration and maturation, respectively (**Figures 2N–R**). At the same time, CALR was knocked down in mCALR cells (**Figures 3A**), and the supernatant of the two siCALR groups inhibited the migration of mDCs compared to the control (**Figures 3B, C**). The percentage of CD83+ DCs significantly decreased in the siCALR groups after incubation with the supernatant compared to that in the controls (**Figures 3D–G**). Likewise, the expressions of TNFα and CCL19 markedly decreased in the siCALR groups (**Figures 3H–K**). These findings indicate that mCALR in NSCLC cells facilitates the migration and maturation of DCs by increasing the secretion of TNFα and CCL19.

### mCALR Enhances the Secretion of TNFα and CCL19 *via* Activating CALR-TLR4-MyD88 Signaling

**Figure 4:**
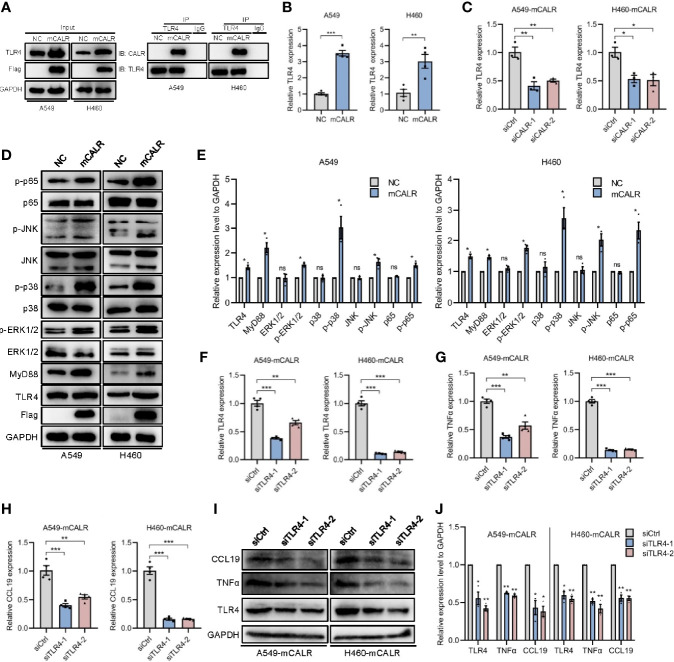
mCALR enhances the secretion of TNFα and CCL19 *via* activating CALR-TLR4-MyD88 signaling. **(A)** The interaction between mCALR and TLR4 was assessed using co-IP assay in NC and mCALR cells, and the IgG antibody was used as a negative control. **(B)** The relative mRNA expression level of TLR4 was detected in NC and mCALR groups using real-time PCR; **P < 0.01, ***P < 0.001. **(C)** The relative mRNA expression level of TLR4 was detected in siCALR and control groups using real-time PCR; *P < 0.05, **P < 0.01. **(D, E)** The expression levels of mCALR, TLR4, MyD88, ERK1/2, p-ERK1/2, p38, p-p38, JNK, p-JNK, p65, and p-p65 were investigated in NC and mCALR groups using western blot, and the anti-Flag antibody was used to detect the mCALR protein; ns, *P*>0.05, *P < 0.05. **(F–H)** The relative mRNA expression levels of TLR4, TNFα, and CCL19 were detected in mCALR cells transfected with different siTLR4 using real-time PCR; **P < 0.01, ***P < 0.001. **(I, J)** The expression levels of TLR4, TNFα, and CCL19 were investigated in mCALR cells transfected with different siTLR4 using western blot; *P < 0.05, **P < 0.01.

**Figure 5:**
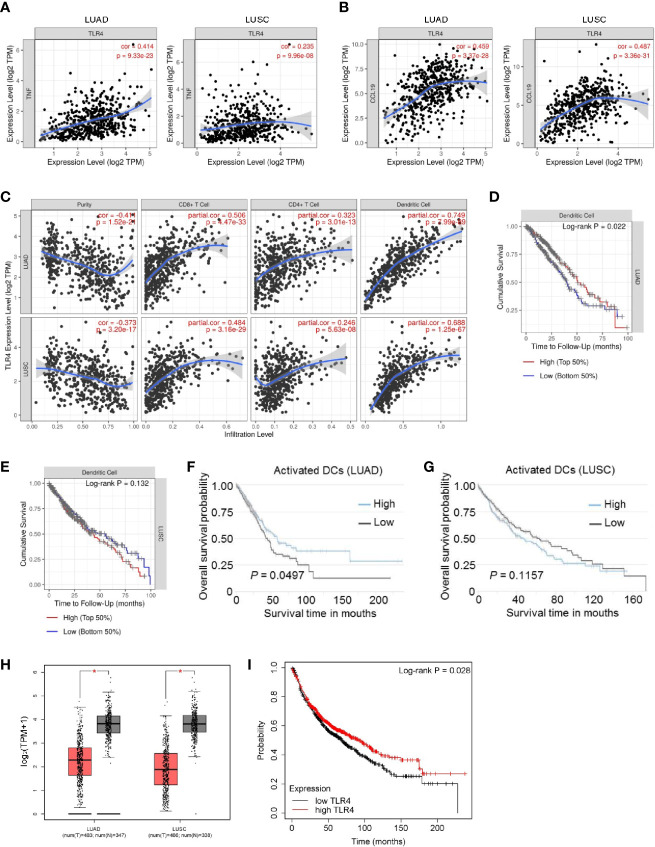
The correlation and overall survival of tumor-infiltrating immune cells in NSCLC patients were analyzed using TIMER and TCIA. **(A)** The correlation of TLR4 and TNFα expressions was analyzed in LUAD and LUSC using TIMER; TPM, Transcripts Per Million. **(B)** The correlation of TLR4 and CCL19 expressions was analyzed in LUAD and LUSC using TIMER; TPM, Transcripts Per Million. **(C)** The correlation of TLR4 expression with immune cell infiltration levels was analyzed in LUAD and LUSC using TIMER. Tumor-infiltrating immune cells included CD8+ T cells, CD4+ T cells, and DCs. Gene expression levels against tumor purity are displayed in the left-most panel; TPM, Transcripts Per Million. **(D, E)** Overall survival rates of patients with high and low DC infiltration were analyzed in LUAD **(D)** and LUSC **(E)** using TIMER; LUAD, 501 patients, *P*=0.022; LUSC, 492 patients, *P*=0.132. **(F, G)** Overall survival rates of patients with high and low activated DC infiltration were analyzed in LUAD **(F)** and LUSC **(G)** using TCIA; LUAD, 506 patients, *P*=0.0497; LUSC, 495 patients, *P*=0.1157. **(H)** The expression of TLR4 in NSCLC (LUAD and LUSC) tissues and normal tissues was analyzed using GEPIA. **(I)** Overall survival rates of patients with high and low TLR4 expression were analyzed in lung cancer (n=1144 patients) using Kaplan-Meier Plotter; *P*=0.028.

TLRs are pattern-recognition receptors that play an important role in mediating the intracellular inflammatory response. Activated TLRs regulate downstream transcription factors through corresponding intracellular signals, causing the secretion of inflammatory factors and chemokines, including TNFα and CCL19 (17, 18). Activation of TLR4 signaling inhibits the progression of osteosarcoma by stimulating CD8+ cytotoxic lymphocytes (19). In view of the above-mentioned facts, we wondered whether mCALR promotes the secretion of TNFα and CCL19 by activating TLR4. Therefore, we investigated the relationship between mCALR and TLR4. The co-IP assay also demonstrated an interaction between mCALR and TLR4, while no interaction was detected between cytoplasmic CALR and TLR4 (**Figure 4A**). Furthermore, TLR4 level was significantly elevated in the mCALR group and decreased in the siCALR groups compared to that of their corresponding controls (**Figures 4A–C**). The mCALR protein activated TLR4-MyD88 signaling and promoted the phosphorylation of ERK1/2, p38, JNK, and p65 (**Figures 4D, E**). Meanwhile, TLR4 was silenced in mCALR cells (**Figure 4F**), and TNFα and CCL19 levels significantly decreased in TLR4 knockdown cells (**Figures 4G–J**). These findings indicate that mCALR enhances the secretion of TNFα and CCL19 by activating CALR-TLR4-MyD88 signaling. Interestingly, TIMER data also showed a significant positive correlation between TLR4 and TNFα expressions in LUAD (cor=0.414, *P*=9.33e-23) and LUSC (cor=0.235, *P*=9.96e-08) (**Figure 5A**). A marked positive correlation between TLR4 and CCL19 expressions was also observed in LUAD (cor=0.459, *P*=3.37e-28) and LUSC (cor=0.487, *P*=3.36e-31) (**Figure 5B**). Moreover, the relationships between TLR4 expression and the infiltration of CD8+ T cells, CD4+ T cells, and DCs were assessed using TIMER against NSCLC datasets. The analysis showed a marked positive correlation between TLR4 expression in tumor cells and the infiltration of CD8+ T cells (partial.cor=0.506, *P*=4.47e-33; partial.cor=0.484, *P*=3.16e-29), CD4+ T cells (partial.cor=0.323, *P*=3.01e-13; partial.cor=0.246, *P*=5.63e-08), and DCs (partial.cor=0.749, *P*=7.99e-89; partial.cor=0.688, *P*=1.25e-67) in LUAD and LUSC, respectively (**Figure 5C**). Meanwhile, a high infiltration level of DCs was significantly associated with good survival in LUAD (**Figures 5D, F**), while no significant difference was observed in LUSC (**Figures 5E, G**). GEPIA data showed that TLR4 was down-regulated in LUAD and LUSC tissues compared to normal tissues (**Figure 5H**). High expression level of TLR4 was significantly associated with good survival in lung cancer (*P*=0.028) (**Figure 5I**). These results further demonstrate that elevated TLR4 activation by mCALR enhances the secretion of TNFα and CCL19 and facilitates DC infiltration in NSCLC tissues, which may contribute to good prognosis of NSCLC patients, especially for LUAD patients.

### mCALR Inhibits Lung Cancer Progression by Facilitating DC Infiltration in Tumor Tissues

**Figure 6:**
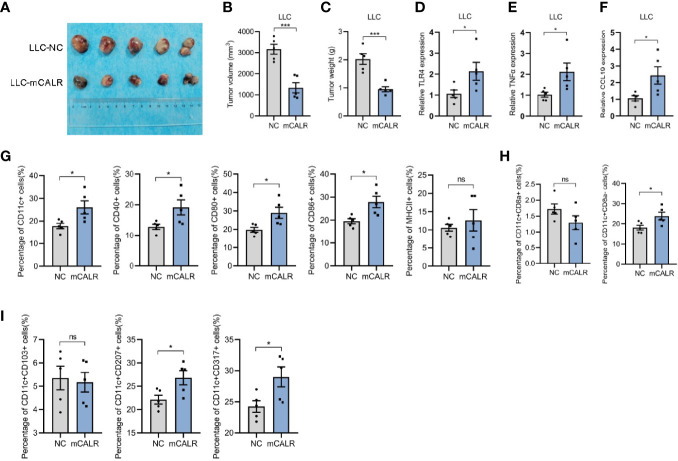
mCALR inhibits lung cancer progression by facilitating DC infiltration in tumor tissues. **(A)** A tumor formation assay was performed by injecting LLC-NC and LLC-mCALR cells, n = 5 mice. **(B, C)** Quantification of the volume **(B)** and weight **(C)** of tumors generated in mice, ***P < 0.001. **(D–F)** The relative mRNA expression levels of TLR4, TNFα, and CCL19 were detected in tumors of LLC-NC and LLC-mCALR groups using real-time PCR; *P < 0.05. **(G)** The relative percentages of CD11c+, CD40+, CD80+, CD86+, and MHCII+ DCs in tumors were detected using flow cytometry; ns, *P* > 0.05, *P < 0.05. **(H)** The relative percentages of CD11c+CD8a+ and CD11c+CD8a- DCs in tumors were detected using flow cytometry; ns, *P* > 0.05, *P < 0.05. **(I)** The relative percentages of CD11c+CD103+, CD11c+CD207+, and CD11c+CD317+ DCs in tumors were detected using flow cytometry; ns, *P* > 0.05, *P < 0.05.

To investigate the role of mCALR in the progression of lung cancer, *in vivo* experiments were conducted. The Lewis cell line with stable expression of CALR on the cell membrane (LLC-mCALR) was constructed by transfection with CALR(del_KDEL)-GPI construct and identified by membrane protein extraction (**Figure S1C**). CCK8 assay showed that mCALR had little effect on LLC cell proliferation (**Figure S2A**). Subsequently, LLC-mCALR and control cells were injected subcutaneously into the C57BL/6J mice. Two weeks later, the tumor masses were removed from the mice (**Figure 6A**). Tumor size was lower in the mCALR group than in the control group (**Figure 6B**). The tumor weight was also lower in the mCALR group than in the control group (**Figure 6C**). The expression of TLR4, TNFα, and CCL19 in tumors was evaluated, and the results showed that their expression levels increased dramatically in the mCALR group (**Figures 6D–F**). Meanwhile, the percentage of CD11c+, CD40+, CD80+, and CD86+ DCs significantly increased in the mCALR group, while no significant change was observed in the percentage of MHCII+ DCs (**Figure 6G**), suggesting that mCALR expression was closely associated with DC infiltration in tumor tissues. Batf3-dependent type 1 conventional DCs plays a critical role in anti-tumor immunity (20), including CD8+ DCs (non-migrating resident DCs) and CD8- DCs (migratory DCs) (21). The results revealed that the number of CD11c+CD8a- DCs markedly increased in mCALR group compared to the control, while the number of CD11c+CD8a+ DCs showed no significant changes (**Figure 6H**). Although no significance was observed in the percentage of CD11c+CD103+ migratory DCs between mCALR and control groups, the percentages of CD11c+CD207+ migratory DCs and CD11c+CD317+ mature DCs were elevated in mCALR group (**Figure 6I**). These findings suggested that mCALR facilitated the migration and maturation of DCs *in vivo*, thereby inhibiting lung cancer progression.

## Discussion

Currently, malignant tumors pose a serious threat to human health, especially in lung cancer. Radiation and chemotherapy are important strategies for tumor treatment. They can be used as neoadjuvant therapy to reduce the size of tumors and ensure follow-up surgery (22). On the other hand, they also serve as adjuvant therapy to reduce the risk of relapse and improve survival (23). Radiochemotherapy induces transformation of tumor cells from non-immunogenic cells to immunogenic cells, thus stimulating the anti-tumor immune response (24).

CALR, a characteristic antigen expressed on tumor cells undergoing ICD, is involved in the anti-tumor immune response. Expose to wildtype CALR in cancer cells enhances the DC efferocytosis of cancer cells, which triggers the activation of immune responses in the tumor microenvironment (25, 26). In our study, we found that the expression of mCALR was positively related to DC infiltration in NSCLC tissues and that mCALR on tumor cells facilitated the migration and maturation of DCs. A previous study showed that overexpression of CALR promoted maturation of DCs, which induced cytotoxic T lymphocyte (CTL) development and enhanced MAGE-A3-specific CTL cytotoxicity against NSCLC (27). Our findings illustrate the role of CALR on the NSCLC cell membrane surface in promoting DC maturation, which provides insights on the role of CALR in anti-tumor immunity. It has been reported that CALR expression in tumor cells correlates with eIF2α phosphorylation and DC infiltration, which positively influences the clinical outcome of NSCLC (28). Our study shows that high levels of mCALR expression and DC infiltration are associated with good prognosis in patients with NSCLC, consistent with a previous study.

DCs are the most effective antigen-presenting cells that play an important role in mediating anti-cancer immune response (29). TLRs are transmembrane receptors with the function of pathogen recognition, participating in immune response and mediating intracellular inflammatory response. Activated TLR signals cause host cells to produce a large number of inflammatory factors by stimulating downstream transcription factors. During chemotherapy or radiotherapy, DCs require signaling through TLR4 and its adaptor MyD88 for efficient processing and cross-presentation of antigens from dying tumor cells. TLR4 expressed by DCs facilitates the activation of tumor-specific T cell immunity (30). Soluble recombinant CART/39-272 is a potent stimulatory agent for DC maturation in the TLR4/CD14 and PI3K/Akt dependent pathway, which initiates cellular immunity and T cell response (31). Our study shows that mCALR on NSCLC cells interacts with its own receptor TLR4, which activates TLR4-MyD88 signaling and promotes the phosphorylation of ERK1/2, p38, JNK, and p65. Subsequently, the secretion of TNFα and CCL19 induces the infiltration and maturation of DCs in tumor tissues, thus inhibiting NSCLC progression. Therefore, TLR4 activation in DCs and tumor cells exhibits a good anti-tumor immune response. These findings provide a theoretical basis for the research and development of tumor vaccines using recombinant CALR.

With the continuous development of tumor immunology, immunotherapy approaches are being increasingly used in cancer treatment, either alone or in combination with chemotherapy or radiotherapy (32). Based on the efficiency and safety, appropriate integration of radiochemotherapy and immunotherapy may increase the therapeutic effect on tumors. We can strengthen the anti-tumor immune response by enhancing the immunogenicity of tumor cells, which provides new strategies and approaches for tumor immunotherapy.

## Conclusion

**Figure 7:**
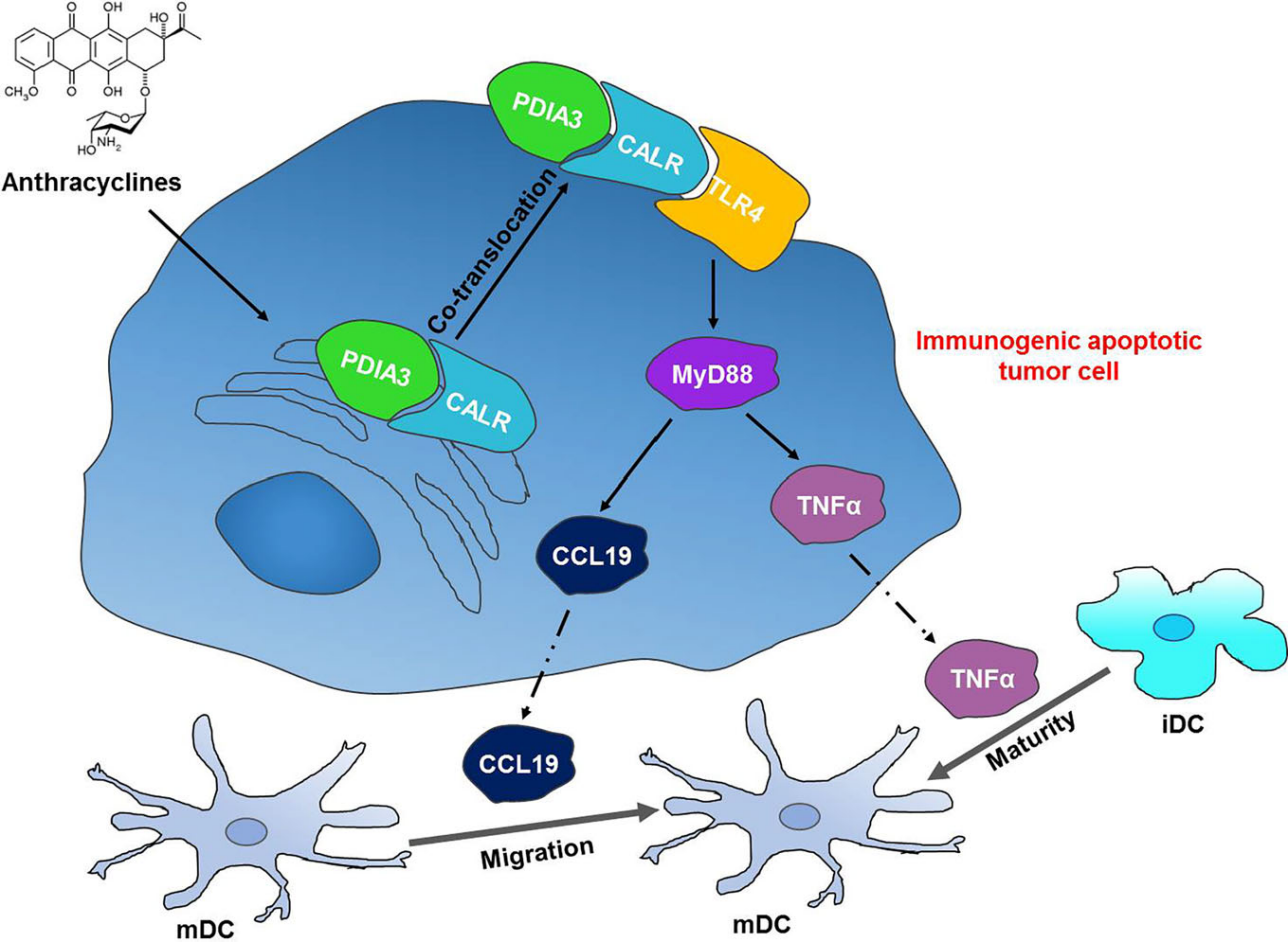
The CALR-TLR4 complex inhibits NSCLC progression by regulating DC migration and maturation and activating anti-tumor immune response. Anthracycline-treated tumor cells form a co-translocation of CALR and PDIA3 from endoplasmic reticulum to cell surface, which causes the interaction between CALR and TLR4. The interaction activates TLR4-MyD88 signaling and promotes the secretion of TNFα and CCL19 and DC infiltration and maturation in the tumor tissues, thereby inducing the immunogenic cell death in NSCLC.

In conclusion, we discovered a novel interaction between mCALR and TLR4 in NSCLC cells, which enhanced the secretion of TNFα and CCL19 *via* activation of TLR4-MyD88 signaling. In addition, the secretion of TNFα and CCL19 fortifies DC migration and maturation in NSCLC tissues and exhibits a good prognosis in patients with NSCLC. Our study demonstrates the inhibitory effect of the CALR-TLR4 complex on NSCLC progression and provides a theoretical basis for NSCLC immunotherapy (**Figure 7**).

## Data Availability Statement

The original contributions presented in the study are included in the article/**Supplementary Material**. Further inquiries can be directed to the corresponding authors.

## Ethics Statement

The studies involving human participants were reviewed and approved by the ethics committee of Tangdu Hospital, Fourth Military Medical University. The patients/participants provided their written informed consent to participate in this study. The animal study was reviewed and approved by the Institutional Animal Care and Use Committee of Fourth Military Medical University.

## Author Contributions

KW and Z-NC conceived the study. All the authors performed the experiments and analyzed the data. RC, MH, and KW wrote the original draft of the manuscript. KW and Z-NC edited the manuscript and supervised the study. All authors contributed to the article and approved the submitted version.

## Funding

This work was supported by a grant from the National Natural Science Foundation of China (82002425) and the Young Talent Fund of China Association for Science and Technology.

## Conflict of Interest

The authors declare that the research was conducted in the absence of any commercial or financial relationships that could be construed as a potential conflict of interest.

## Publisher’s Note

All claims expressed in this article are solely those of the authors and do not necessarily represent those of their affiliated organizations, or those of the publisher, the editors and the reviewers. Any product that may be evaluated in this article, or claim that may be made by its manufacturer, is not guaranteed or endorsed by the publisher.

## Supplementary Material

Supplementary Figure 1 Identification of cell lines with stable expression of CALR on the cell membrane. **(A, B, C)** The expression of Flag-CALR in mCALR cells (A549, H460, and LLC) was investigated by isolation of membrane and cytoplasmic proteins, and the levels of membrane and cytoplasmic proteins were normalized to the expression levels of NA+/K+-ATPase and GAPDH, respectively.

Supplementary Figure 2 mCALR has no effect on the proliferation and migration of lung cancer cells. **(A)** Proliferation ability of A549-mCALR, H460-mCALR, LLC-mCALR, and their controls was evaluated by CCK8 assay; ns, *P* > 0.05. **(B)** Migration ability of A549-mCALR, H460-mCALR, and their controls was evaluated by migration assay, ns, *P* > 0.05.

Supplementary Figure 3 The induction and identification of iDC and mDC. **(A)** The schematic diagram for *in vitro* iDC and mDC induction. **(B)** The identification of iDC and mDC by flow cytometry.
