# Supplementary figures and images for: CALR-TLR4 Complex Inhibits Non-Small Cell Lung Cancer Progression by Regulating the Migration and Maturation of Dendritic Cells
Source: Front Oncol. 2021 Oct 1;11:743050. doi: 10.3389/fonc.2021.743050 (PMC8517398; doi:10.3389/fonc.2021.743050)

Fig. S1

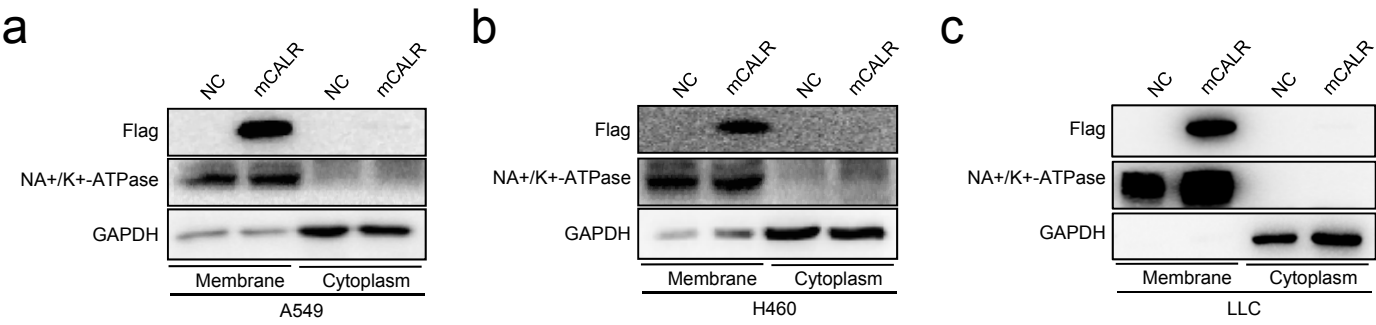

Fig. S2

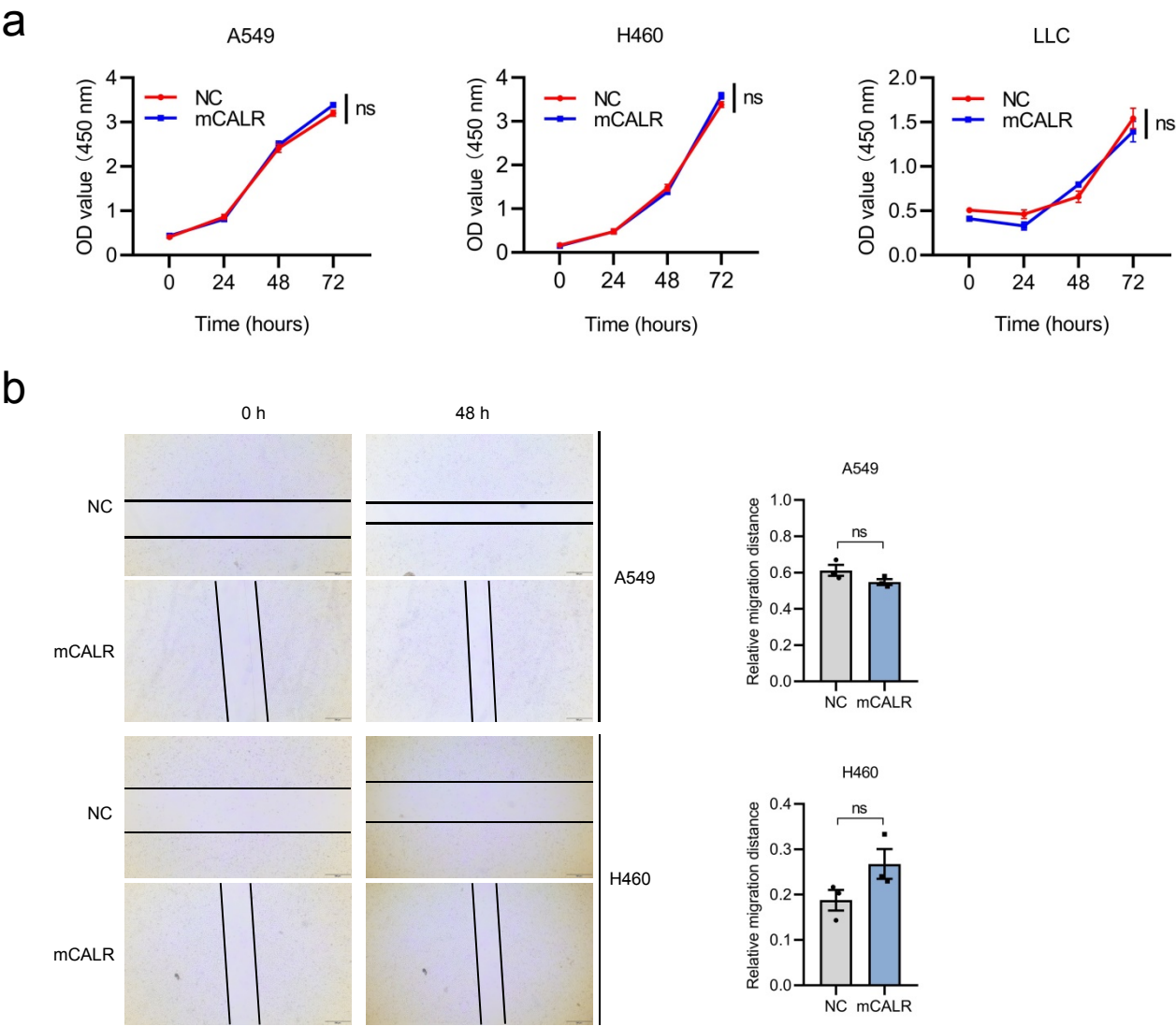

Fig. S3

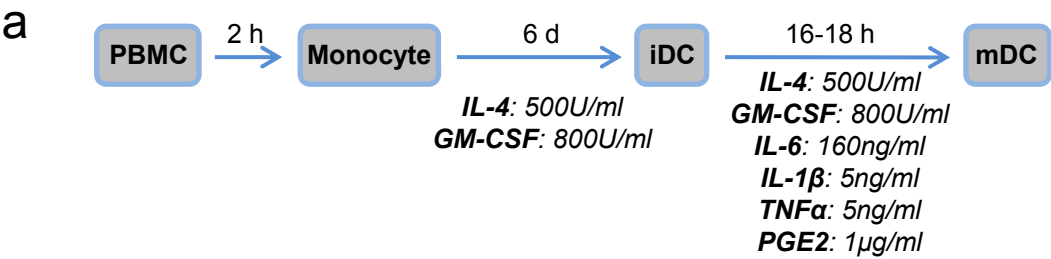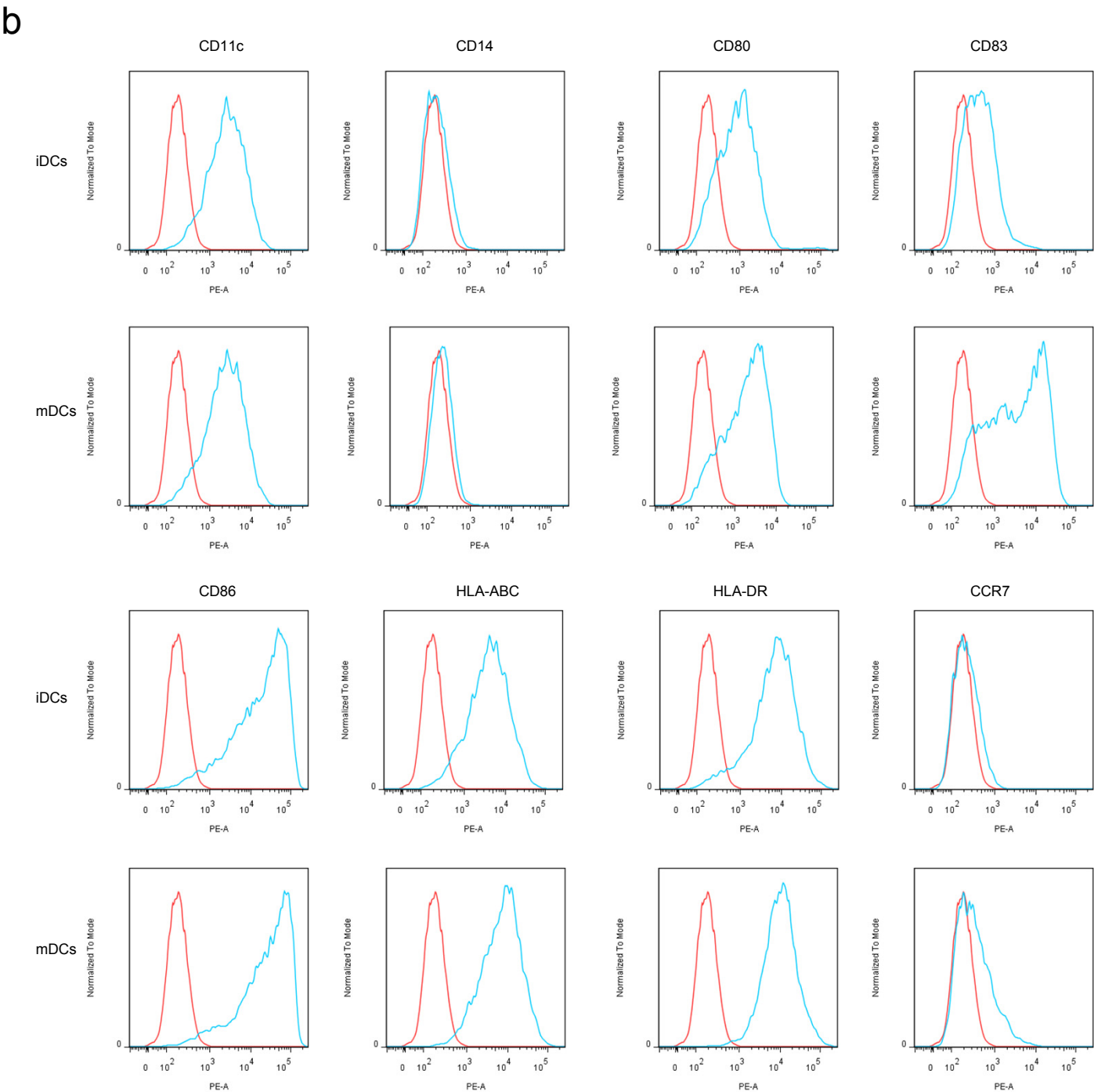

Supplement: Supplementary Figure 1 — Identification of cell lines with stable expression of CALR on the cell membrane. (A, B, C) The expression of Flag-CALR in mCALR cells (A549, H460, and LLC) was investigated by isolation of membrane and cytoplasmic proteins, and the levels of membrane and cytoplasmic proteins were normalized to the expression levels of NA+/K+-ATPase and GAPDH, respectively. [file DataSheet_1.pdf]
