# Supplementary material for: CALR-TLR4 Complex Inhibits Non-Small Cell Lung Cancer Progression by Regulating the Migration and Maturation of Dendritic Cells
Source: Front Oncol. 2021 Oct 1;11:743050. doi: 10.3389/fonc.2021.743050 (PMC8517398; doi:10.3389/fonc.2021.743050)
Supplement: Supplementary file 2 [file Table_1.docx]

**Table S1. The primers for targeted genes in real-time PCR**

Human GAPDH-Forward: GCACCGTCAAGGCTGAGAAC

Human GAPDH-Reverse: TGGTGAAGACGCCAGTGGA

Human CALR-Forward: CGAGCCTTTCAGCAACA

Human CALR-Reverse: CAGACTTGACCTGCCAGAG

Human TLR4-Forward: AGTTGATCTACCAAGCCTTGAGT

Human TLR4-Reverse: GCTGGTTGTCCCAAAATCACTTT

Human TNFα-Forward: GAGGCCAAGCCCTGGTATG

Human TNFα-Reverse: CGGGCCGATTGATCTCAGC

Human CCL19-Forward: TACATCGTGAGGAACTTCCACT

Human CCL19-Reverse: CTGGATGATGCGTTCTACCCA

Mouse Actin-Forward: AGATTACTGCTCTGGCTCCTAGC

Mouse Actin-Reverse: ACTCATCGTACTCCTGCTTGCT

Mouse TLR4-Forward: ATGGCATGGCTTACACCACC

Mouse TLR4-Reverse: GAGGCCAATTTTGTCTCCACA

Mouse TNFα-Forward: CAGGCGGTGCCTATGTCTC

Mouse TNFα-Reverse: CGATCACCCCGAAGTTCAGTAG

Mouse CCL19-Forward: GGGGTGCTAATGATGCGGAA

Mouse CCL19-Reverse: CCTTAGTGTGGTGAACACAACA
