# Supplementary material for: CALR-TLR4 Complex Inhibits Non-Small Cell Lung Cancer Progression by Regulating the Migration and Maturation of Dendritic Cells
Source: Front Oncol. 2021 Oct 1;11:743050. doi: 10.3389/fonc.2021.743050 (PMC8517398; doi:10.3389/fonc.2021.743050)
Supplement: Supplementary file 3 [file Table_2.docx]

**Table S2. The sequences of siRNA**

siCtrl sense: 5’-UUCUCCGAACGUGUCACGUTT-3’

siCtrl antisense: 5’-ACGUGACACGUUCGGAGAATT-3’

siTLR4-1 sense: 5’-CCCACAUUGAAACUCAAAUTT-3’

siTLR4-1 antisense: 5’-AUUUGAGUUUCAAUGUGGGTT-3’

siTLR4-2 sense: 5’-GGGCUUAGAACAACUAGAATT-3’

siTLR4-2 antisense: 5’-UUCUAGUUGUUCUAAGCCCTT-3’

siCALR-1 sense: 5’-GCUGGAUCGAAUCCAAACATT-3’

siCALR-1 antisense: 5’-UGUUUGGAUUCGAUCCAGCTT-3’

siCALR-2 sense: 5’-CUUGGAUCCACCCAGAAAUTT-3’

siCALR-2 antisense: 5’-AUUUCUGGGUGGAUCCAAGTT-3’
